# Association between Pregnancy Loss and Urinary Phthalate Levels around the Time of Conception

**DOI:** 10.1289/ehp.1103552

**Published:** 2011-11-23

**Authors:** Gunnar Toft, Bo A.G. Jönsson, Christian H. Lindh, Tina Kold Jensen, Niels H. Hjollund, Anne Vested, Jens Peter Bonde

**Affiliations:** 1 Danish Ramazzini Center, Department of Occupational Medicine, Aarhus University Hospital, Aarhus, Denmark; 2 Division of Occupational and Environmental Medicine, Department of Laboratory Medicine, Lund University Hospital, Lund, Sweden; 3 Department of Environmental Medicine, University of Southern Denmark, Odense, Denmark; 4 Department of Occupational Medicine, Herning Regional Hospital, Herning, Denmark; 5 Department of Clinical Epidemiology, Aarhus University Hospital, Aarhus, Denmark; 6 Department of Occupational and Environmental Medicine, Copenhagen University Hospital Bispebjerg, Copenhagen, Denmark

**Keywords:** abortion, MEHP, phthalates, pregnancy loss

## Abstract

Background: Animal studies indicate that some phthalate metabolites may harm female reproductive function.

Objectives: We assessed the associations between exposure to phthalate metabolites and pregnancy loss.

Methods: Using a previously established cohort of couples planning their first pregnancy, we analyzed four primary and two oxidized secondary phthalate metabolites in urine samples collected on day 10 after the first day of the last menstrual period before conception occurred (*n* = 128) and during the previous cycle (if any, *n* = 111). Subclinical embryonal loss was identified by repeated measurement of urinary human chorionic gonadotropin, and information on clinical spontaneous abortions was obtained by telephone interview with the mother.

Results: Pregnancy loss (*n* = 48) was increased among women with urinary concentration of monoethylhexyl phthalate (MEHP) in the upper tertile in the conception sample compared with women in the lowest tertile [adjusted odds ratio (OR) = 2.9; 95% confidence interval (CI): 1.1, 7.6]. The corresponding OR for subclinical embryonal loss (*n* = 32) was 40.7 (95% CI: 4.5, 369.5).

Conclusions: The phthalate metabolite MEHP was associated with higher occurrence of pregnancy loss. Because this is the first human study to show this association and the sample size is small, the findings need to be corroborated in independent studies.

Phthalates including di-(2-ethylhexyl)phthalate (DEHP) and di-isononyl phthalate are added to polyvinyl plastic materials to increase flexibility. Phthalates can be released into food during processing and from packing material, with food believed to be the main source of human exposure (Wormuth et al. 2006). In addition, a number of cosmetic compounds contain diethyl phthalate (DEP) and/or di-*n*-butyl phthalate (DBP). These phthalates can cross the human skin after application and thereby represent an additional source of exposure (Janjua et al. 2008). Several million tons of phthalates are used worldwide every year (Wormuth et al. 2006).

The elimination half-life of phthalate metabolites from the human body is relatively fast, with a half-life of < 24 hr for most metabolites (Koch et al. 2006). However, because of continuous exposure, phthalates can be detected in almost all studied human samples. In Germany, the estimated phthalate intake has been reported to exceed acceptable daily intake for about 14% of the population (Wittassek et al. 2007).

Rodent studies indicate that exposure to specific phthalates may induce female reproductive toxicity. In a continuous breeding protocol on mice, a reduction in the number of litters, litter size, and proportion of liveborn offspring was observed after DEHP exposure as well as after exposure to higher concentrations of DBP (Lamb et al. 1987), whereas increased risk of mid-pregnancy abortions was found among DBP-exposed rats in another study (Gray et al. 2006; Lamb et al. 1987). However, the concentrations of phthalate used in these rodent studies were > 100 times higher than the average estimated daily intake for the general population (Frederiksen et al. 2011). So far, phthalate exposure in women has not been associated with measures of fertility. However, an association between increased phthalate level and precocious breast development has been observed (Colon et al. 2000). Moreover, phthalate exposure among adult women has been associated with endometriosis (Cobellis et al. 2003) and gestational duration, although with inconsistent results (Adibi et al. 2009; Latini et al. 2003; Wolff et al. 2008).

In humans, about one-third of all pregnancies terminate before the fetus is fully developed, and about two-thirds of these are lost early in pregnancy at the embryonic stage (Wilcox et al. 1988). Few environmental causes of early pregnancy loss have been established, but recent studies indicate that environmental contaminants [e.g., dichlorodiphenyltrichloroethane (DDT) exposure (Venners et al. 2005)] and lifestyle factors such as alcohol consumption (Henriksen et al. 2004) and physical strain (Hjollund et al. 2000b) may increase the risk of early pregnancy loss. To date, no studies have investigated the association between pregnancy loss and phthalate exposure in human populations.

The extensive use of phthalates together with the possible effects on human health makes it important to develop methods to assess exposure. Phthalates are rapidly hydrolyzed to the biologically active monoesters in the body and excreted in the urine (Wittassek and Angerer 2008). Thus, monoethyl phthalate (MEP) is a marker of DEP, monobenzyl phthalate (MBzP) of benzyl butyl phthalate (BBzP), and monobutyl phthalate (MBP) mainly of DBP and BBzP. Finally, monoethylhexyl phthalate (MEHP) is a marker of DEHP exposure (Anderson et al. 2001). In addition, MEHP is further oxidized to hydroxy-MEHP and oxo-MEHP. Techniques using liquid chromatography–tandem mass spectrometry (LC/MS/MS) have provided rapid methods for measuring these metabolites in urine (Blount et al. 2000).

We examined the association between phthalate exposure during pregnancy attempts and pregnancy loss, including both early biochemical detectable losses and abortions of clinically recognized pregnancies.

## Methods

From 1992 to 1994, a total of 430 couples were enrolled in a Danish prospective cohort study of fecundity after a nationwide mailing of personal letters to > 50,000 members of four trade unions (Bonde et al. 1998). Letters were sent to union members who were 20–35 years of age, lived with a partner of the opposite sex, and had no children. Only couples without any knowledge about their reproductive capacity and who planned to stop contraception to conceive were invited to participate. They were enrolled when birth control was discontinued and followed prospectively until a clinically recognized pregnancy occurred, or for six menstrual cycles if there was no clinical pregnancy. A crude estimate of the participation rate, based on an estimation of the eligible population, has been calculated as 16% (Bonde et al. 1998). The women filled in a diary with menstrual cycle data and produced a urine sample each day of the first 10 days of each menstrual cycle until a pregnancy was achieved, or for a maximum of six cycles. In addition, the women and their partners filled in a detailed questionnaire on occupation and health, and the outcome of each pregnancy was ascertained by an interview after 1 year (Bonde et al. 1998; Hjollund et al. 2000a).

All urine samples from the first day of the vaginal bleeding period were analyzed for the content of human chorionic gonadotropin (hCG) by an immunofluorometric assay (DELFIA, Wallace, Finland). This method is specific for intact hCG and was validated for measurements of hCG in urine. Serial dilutions of urinary samples resulted in a linear response curve parallel to the standard curve. For 12 women not reporting any intercourse for the preceding 2 months, all urinary hCG measurements were below the detection limit of 0.5 IU/L. If the hCG level was > 0.8 IU/L in a sample from the first day after vaginal bleeding, the following nine samples were analyzed as well (Bonde et al. 1998). Early pregnancy loss was considered to have occurred if any of the hCG values from one menstrual cycle was > 1 IU/L, followed by a decline in the hCG level.

**Figure 1:**
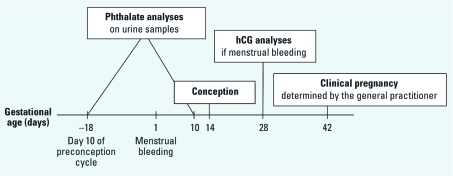
Overview of the timing of selected samples for phthalate analyses in relation to time of conception, hCG analysis, and ascertainment of clinical pregnancy. The times given represent averages in the study population.

In 2009, at least one series of stored urine samples was available for 242 of the 430 women who were enrolled in the pregnancy planner study. In 77 women, no urine samples were collected because conception occurred during the first menstrual cycle. In addition, some samples (*n* = 111) were lost during the storage period for unknown reasons. Age and average amounts of smoking and drinking were very similar between women with and without analyzed samples; but waiting time to pregnancy was shorter and the proportion of couples achieving a pregnancy after 12 months was higher among women without phthalate measurements, because this group included a large proportion of women who became pregnant in the first menstrual cycle (data not shown). From each of the women with available urine samples, we selected up to two urine samples for analysis of phthalate metabolites (Figure 1), including one conception sample from day 10 after the first day of the last menstrual period before their first pregnancy (registered as either a clinical pregnancy or hCG elevation) (*n* = 128), and one preconception sample from day 10 of the previous menstrual period termed, when available (*n* = 111).

The final sample comprised 128 women with a pregnancy during the study period and at least one urinary phthalate analysis available, including 80 who had a pregnancy that ended with the birth of a liveborn child and 48 who had a pregnancy loss, including 32 identified by hCG elevations only. Women who did not become pregnant during the study period (*n* = 114) were not at risk of pregnancy loss and were excluded from the analysis.

Pregnancy loss was defined as the loss of a pregnancy identified based on hCG elevation after menstrual bleeding only (classified as an early pregnancy loss) or the loss of a clinical pregnancy recognized by the woman’s general practitioner (spontaneous abortion or later pregnancy loss).

All participants in the study signed informed consent when enrolled in the study, and the study (including the additional urinary analysis of phthalates) was approved by the ethical committee of the Central Region, Denmark.

*Exposure assessment.* The urine samples were processed using an automated solid-phase extraction technique and analyzed by LC/MS/MS. Briefly, after adding internal standards, the samples were treated with glucuronidase to remove glucuronic acid and acidified, and the metabolites were extracted using Oasis HLB 3 mL (60 mg) on an Aspec XL4 automated solid-phase extraction equipment (Gilson, Middleton, WI, USA). The samples were then evaporated and dissolved in water containing acetic acid. ^2^H_4_-MEP, ^2^H_4_-MBP, ^2^H_4_-MEHP, ^13^C_4_-MBzP, ^13^C_4_-5-hydroxy-MEHP, and ^13^C_4_-5-oxo-MEHP were used as internal standards. The MS parameters, precisions, and detection limits are shown in Supplemental Material, Table 1 (http://dx.doi.org/10.1289/ehp.1103552). Values for MBP and hydroxy-MEPH were within the tolerance limits obtained in samples from a round-robin interlaboratory control program from the University of Erlangen–Nürnberg, Germany. However, measured values deviated from the tolerance limits for oxo-MEHP. A possible explanation for this has been indicated by the Erlangen group: The standards from Cambridge Isotope Laboratories used in the analysis have been described as having up to 60% different concentrations in different batches. No interlaboratory control programs were available for the other compounds. In each series, an internal control urine sample was analyzed, and there was no temporal variation in the measured concentrations of any of the metabolites in the control sample. A hand refractometer was used to determine urine density. To calculate density-adjusted urine concentrations, the following formula was used: C_corr_ = C_obs_ × 0.016/(ρ–1), where C_corr_ is the corrected concentration, C_obs_ is the observed concentration and ρ is the specific density of the urine sample (Boeniger et al. 1993). Except for 15 samples that had MEHP levels below the limit of detection (LOD; 2 ng/mL), all selected phthalate metabolites were detected in all the measured urine samples. The actual readout from the LC/MS/MS was used for values below the LOD.

**Table 1:** Distribution of phthalate metabolites in the cycle where women achieved a pregnancy and covariates among women experiencing loss of an hCG-detected or a clinical pregnancy and women carrying a pregnancy to term.

Phthalate exposure and potential confounders	Pregnancy loss (*n* = 48)	Liveborn child (*n* = 80)	*p*-Value for difference*a*
Phthalate exposure [mean (minimum, maximum)]						
MEP (ng/mL)		377.6 (27.9, 2765.9)		405.8 (19.3, 2782.8)		0.77
MBP (ng/mL)		255.1 (37.7, 955.4)		225.6 (11.8, 1005.0)		0.39
MBzP (ng/mL)		21.1 (2.4, 173.5)		20.3 (0.8, 117.0)		0.87
MEHP (ng/mL)		23.4 (< LOD, 84.0)		16.2 (< LOD, 64.0)		0.01
5-hydroxy-MEHP (ng/mL)		51.9 (9.5, 207.1)		43.2 (3.6, 215.3)		0.18
5-oxo-MEHP (ng/mL)		48.6 (5.7, 245.9)		40.3 (2.7, 222.2)		0.22
Potential confounders [mean (minimum, maximum) or %]						
Age at enrollment in the study.		25.5 (19.0, 32.0)		25.3 (21.0, 35.0)		0.73
Alcohol (drinks/week)		4.2 (0, 25)		5.8 (0, 35)		0.16
Smoking (cigarettes/day)		1.1 (0,17.0)		3.3 (0, 20)		0.04
Caffeine intake (milligrams caffeine/day)		350 (56, 1,006)		280 (0, 1,017)		0.09
BMI < 20.0 kg/m		27		23		0.84
BMI 20.0–25.0		58		61		
BMI > 25.0		15		16		
**a***t*-Test for continuous data and chi-square test for categorical data.						

*Statistical methods.* We estimated the odds ratios (ORs) for pregnancy loss according to urinary phthalate metabolite concentration grouped into tertiles by logistic regression. Results were presented separately according to exposure in the preconception and the conception cycle to evaluate whether associations differed according to the proximity of the exposure measurement to the time of fertilization. To assure the same exposure limits between tertiles in the two sets of analyses, data analyses were based on tertiles calculated from the exposure distribution in the conception cycle.

Furthermore, separate logistic models were used to estimate associations with early pregnancy loss (hCG determined) and later pregnancy loss compared with women who became pregnant and did not experience a pregnancy loss. These analyses were performed to evaluate whether early loss was more sensitive to phthalate exposure than losses occurring later in the pregnancy.

In the logistic regression models, we adjusted for potential confounders suspected to influence female risk of pregnancy loss [age (continuous), body mass index (BMI) (low < 20; medium 20–25, and high > 25 kg/m), smoking (cigarettes/day, continuous; nonsmokers = 0 cigarettes/day), alcohol consumption (units/weeks, continuous), caffeine intake (milligrams caffeine/day, continuous)] and exposure to the individual measured chemicals in the preconception cycle for analyses based on the conception cycle and vice versa (exposure grouped in tertiles). We considered *p* < 0.05 (two-tailed tests) statistically significant. All data analyses were performed with SAS version 9.1 (SAS Institute Inc., Cary, NC, USA).

## Results

Exposure distributions for the measured phthalate metabolites in the conception cycle are shown in Table 1. Among all women in the study, we found the highest exposure to MEP ranging from 19.3 to 2782.8 ng/mL, followed by MBP (11.8–1005.0 ng/mL), which is considerably higher than values for the secondary MEHP metabolites 5-oxo-MEHP (2.7–245.9 ng/mL) and 5-hydroxy-MEHP (3.6–215.3 ng/mL), followed by MBzP (0.8–173.5 ng/mL) and MEHP (< LOD–84 ng/mL). Of 15 MEHP samples < LOD, 6 were found on samples from the conception cycle (two from women with pregnancy loss and four from women not experiencing a pregnancy loss). The correlation between samples from the conception and preconception cycle was 0.40 for MEP, 0.28 for 5-oxo-MEHP, 0.27 for 5-hydroxy-MEHP, 0.20 for MBP, 0.17 for MBzP, and 0.08 for MEHP.

The average MEHP exposure in the conception cycle was significantly different between women experiencing a pregnancy loss and those who did not experience a pregnancy loss [23.4 ng/mL (range, < LOD–84.0) vs. 16.2 ng/mL (range, < LOD–64.0)], whereas the other measured phthalate metabolites did not differ significantly (Table 1). On average, women who had a pregnancy loss smoked fewer cigarettes per day and had a tendency toward higher caffeine consumption than did women without a pregnancy loss, but other potential confounders were similar between the outcome groups. The distributions of potential confounders were similar according to MEHP exposure tertiles in the conception cycle [see Supplemental Material, Table 2 (http://dx.doi.org/10.1289/ehp.1103552)] except for the average number of cigarettes smoked, which was the highest in the middle tertile (4.4/day vs. 1.4 and 1.5/day in the lowest and highest tertiles, respectively).

**Table 2:** ORs (95% CIs) for pregnancy loss according to tertiles of density-adjusted spot urine concentration of phthalate metabolites in the menstrual cycle preceding a conception (preconception cycle) and in the cycle where women achieved a pregnancy (conception cycle).

First tertile			Second tertile					Third tertile				
Phthalate metabolite	*n* (% loss)*a*	OR	*n* (% loss)*a*	OR crude	OR adj*b*	*n* (% loss)*a*	OR crude	OR adj*b*
Preconception cycle																
MEP		18 (44)		1		12 (39)		0.81 (0.31, 2.09)		0.93 (0.35, 2.50)		14 (36)		0.72 (0.29, 1.76)		0.81 (0.31, 2.09)
MBP		17 (49)		1		13 (33)		0.51 (0.20, 1.30)		0.70 (0.27, 1.84)		14 (39)		0.67 (0.26, 1.73)		0.79 (0.32, 2.00)
MBzP		9 (43)		1		22 (48)		1.22 (0.43, 3.46)		1.38 (0.53, 3.62)		13 (30)		0.56 (0.19, 1.65)		0.59 (0.21, 1.65)
MEHP		23 (45)		1		14 (30)		0.53 (0.23, 1.23)		0.74 (0.30, 1.82)		7 (54)		1.42 (0.42, 4.82)		1.79 (0.47, 6.81)
5-hydroxy-MEHP		16 (48)		1		15 (43)		0.84 (0.32, 2.19)		1.33 (0.51, 3.40)		13 (30)		0.46 (0.17, 1.18)		0.57 (0.22, 1.48)
5-oxo-MEHP		17 (43)		1		18 (51)		1.43 (0.58, 3.56)		2.01 (0.80, 5.04)		9 (25)		0.45 (0.17, 1.20)		0.46 (0.16, 1.34)
Conception cycle																
MEP		14 (33)		1		16 (37)		1.46 (0.60, 3.57)		1.51 (0.57, 3.98)		18 (42)		1.61 (0.66, 3.92)		1.98 (0.74, 5.34)
MBP		15 (36)		1		16 (37)		1.07 (0.44, 2.58)		1.12 (0.41, 3.02)		17 (40)		1.18 (0.49, 2.83)		1.12 (0.43, 2.95)
MBzP		13 (31)		1		17 (40)		1.56 (0.60, 3.57)		1.72 (0.63, 4.69)		18 (42)		1.61 (0.66, 3.92)		2.10 (0.74, 5.88)
MEHP		13 (31)		1		12 (28)		0.86 (0.34, 2.20)		0.97 (0.34, 2.76)		23 (53)		2.57 (1.06, 6.23)		2.87 (1.09, 7.57)
5-hydroxy-MEHP		15 (36)		1		14 (33)		0.87 (0.35, 2.13)		0.94 (0.34, 2.59)		19 (44)		1.43 (0.60, 3.41)		1.46 (0.56, 3.78)
5-oxo-MEHP		14 (33)		1		17 (40)		1.31 (0.54, 3.17)		1.22 (0.46, 3.27)		17 (40)		1.31 (0.54, 3.18)		1.38 (0.51, 3.74)
Tertiles are based on exposure distribution in the conception cycle.**a**Loss of an hCG-detected or clinical pregnancy in this tertile (percentage loss/all pregnancies). **b**Adjusted (adj) for age, BMI, smoking, alcohol consumption, caffeine consumption, and exposure to the specific compound analyzed in the preconception cycle for the analyses based on the conception cycle and vice versa.																

The association of measured phthalate exposure in the preconception cycle with pregnancy loss was not statistically significant (Table 2). However, MEHP in the conception sample was significantly associated with pregnancy loss [adjusted OR = 2.87; 95% confidence interval (CI): 1.09, 7.57 for the third tertile compared with the first tertile, *p* = 0.03)] (Table 2). Crude ORs were generally consistent with adjusted ORs. The highest tertiles of all of the other measured phthalate metabolites in the conception samples were also associated with pregnancy loss, but ORs were not significant.

Of the 48 pregnancy losses included in this study, 32 were detected by hCG elevation before gestational week 6, whereas the remaining 16 were lost later in the pregnancy. When we analyzed early and later pregnancy loss separately, early pregnancy loss was increased for the third tertile of MEHP exposure in the conception sample compared with the first tertile, with only one case of early pregnancy loss at the lowest MEHP tertile (OR = 40.67; CI: 4.48, 369.50); late pregnancy loss was inversely associated with MEHP (OR = 0.25; CI: 0.05, 1.8 and OR = 0.17; CI: 0.03, 0.95 for the third and second tertiles compared with the first, respectively) based on two cases in the second and the third tertile (Table 3). None of the other phthalate metabolites were statistically significantly associated with early or late pregnancy loss in the separate analysis, but several were positively associated with early pregnancy loss.

**Table 3:** Analysis of MEHP divided into separate analysis of early embryonal loss and later pregnancy loss in the cycle where women achieved a pregnancy [OR (95% CI)].

First tertile			Second tertile					Third tertile				
Phthalate exposure	*n* (% loss)*a*	OR	*n* (% loss)*a*	OR crude	OR adj*b*	*n* (%) loss*a*	OR crude	OR adj*b*
Early pregnancy loss																
MEP		12 (26)		1		12 (32)		1.34 (0.50, 3.61)		1.29 (0.43, 3.83)		10 (29)		1.16 (0.42, 3.23)		1.13 (0.36, 3.59)
MBP		9 (25)		1		10 (27)		1.11 (0.39, 3.17)		1.25 (0.38, 4.09)		13 (33)		1.50 (0.54, 4.10)		1.64 (0.52, 5.20)
MBzP		7 (19)		1		12 (32)		1.91 (0.66, 5.59)		2.39 (0.70, 8.22)		13 (34)		2.15 (0.74, 6.24)		3.11 (0.87, 11.09)
MEHP		1 (3)		1		10 (24)		9.36 (1.13, 77.71)		10.83 (1.16, 101.48)		21 (51)		30.45 (3.78, 245.06)		40.67 (4.48, 369.50)
5-hydroxy-MEHP		8 (23)		1		10 (26)		1.16 (0.40, 3.38)		1.05 (0.31, 3.56)		14 (37)		1.97(0.70, 5.50)		2.12 (0.67, 6.67)
5-oxo-MEHP		6 (18)		1		13 (33)		2.33 (0.77, 7.04)		2.16 (0.63, 7.39)		13 (33)		2.33 (0.77, 7.04)		2.73 (0.78, 9.54)
Loss of clinical pregnancy																
MEP		3 (11)		1		5 (16)		1.86 (0.40, 8.55)		2.19 (0.42, 11.45)		8 (24)		3.09 (0.74, 12.93)		4.63 (0.92, 23.26)
MBP		6 (18)		1		6 (18)		1.00 (0.29, 3.49)		0.87 (0.21, 3.57)		4 (13)		0.69 (0.18, 2.74)		0.51 (0.12, 2.21)
MBzP		6 (17)		1		5 (16)		0.93 (0.25, 3.41)		1.08 (0.25, 4.66)		5 (17)		0.97 (0.26, 3.55)		0.96 (0.20, 4.59)
MEHP		12 (29)		1		2 (6)		0.16 (0.03, 0.76)		0.17 (0.03, 0.95)		2 (9)		0.24 (0.05, 1.20)		0.25 (0.05, 1.28)
5-hydroxy-MEHP		7 (21)		1		4 (12)		0.53 (0.14, 2.02)		0.91 (0.19, 4.16)		5 (17)		0.80 (0.23, 2.87)		0.90 (0.23, 3.61)
5-oxo-MEHP		8 (22)		1		4 (13)		0.54 (0.15, 2.00)		0.55 (0.13, 2.29)		4 (13)		0.54 (0.15, 2.00)		0.55 (0.13, 2.35)
Tertiles are based on exposure distribution in the conception cycle.**a**Loss in this tertile (percentage loss/all pregnancies). **b**Adjusted (adj) for age, BMI, smoking, alcohol consumption, caffeine consumption, and exposure to the specific compound analyzed in the preconception cycle for the analyses based on the conception cycle and vice versa.																

## Discussion

In our study we found a statistically significant association between elevated periconceptional MEHP exposure and pregnancy loss. Furthermore, subanalyses found that early loss of a pregnancy was more frequent at higher MEHP exposure, although because of a limited number in this group, the estimated OR was associated with a wide CI. The remarkably low incidence of early pregnancy loss (3%) in the low MEHP exposure group suggests that the highly elevated OR for early pregnancy loss may be attributable, at least partly, to chance findings. Pregnancy loss was not significantly associated with other metabolites in the conception sample or with any of the phthalate metabolites in the preconception sample.

Determining loss of a pregnancy by hCG elevation is considered very accurate. Urine samples for hCG analysis were missing in 21% of the cycles, mainly for logistical reasons. Because of missing sample collection, some early pregnancy losses might not have been identified. However, this is not likely to be associated with either exposure or probability of early pregnancy loss. Clinical pregnancies were determined by the general practitioner 2 weeks, on average, after a missed menstrual period, and information on the outcome of the pregnancy was collected for all reported clinical pregnancies. Therefore, we do not suspect misclassification of the clinical pregnancies.

Participants in our study had no prior knowledge about their fecundity, because only first pregnancy planners who had used contraception were included. Thus, it is unlikely that couples with known problems related to fertility were selected. Even if the included cohort was different from the background population in relation to fecundity, the participants had no knowledge about phthalate exposure and were probably not taking any precautions to avoid these chemicals, as the potential harmful effects of phthalates were not discussed in the public in the early 1990s when couples were enrolled for the present study. Therefore, selection bias is unlikely. Although the participation rate was only approximately 16% and our study population was not a representative sample of the general population (Bonde et al. 1998), we have no reason to believe that the associations between phthalate exposure and pregnancy loss would be different in another sample of female pregnancy planners. The exclusion in the present study of highly fertile couples achieving a pregnancy in the first menstrual cycle after discontinuation of contraception, where urine samples were not collected before pregnancy occurred, limited the external validity of the study, and if the highly fecund women were less sensitive to phthalate exposure, our results may be an overestimate of the risk for the general population. Also, because of the design of our study, we were not able to evaluate the association between phthalate exposure and pregnancy loss on the subfecund couples who did not achieve a pregnancy within the study period of 6 months, which might underestimate the effects in the general population if the subfecund couples were more sensitive to phthalate exposure.

An unexpected finding was an inverse association between MEHP exposure and late pregnancy loss (Table 3). This might be explained by a strong effect of MEHP on early loss leading to elimination of vulnerable pregnancies that would be aborted later because of other causes. This phenomenon of apparently protective effects on later reproductive events of exposures with adverse effects on early outcomes is well known in reproductive toxicology (Selevan and Lemasters 1987). Alternatively, it may be speculated that misclassification of etiologically relevant exposure is larger for loss of clinical pregnancies if the losses are induced by exposure later in pregnancy. This would make the study less able to detect associations between exposure and loss of clinical pregnancies and could explain, in part, the lack of a positive association with clinical pregnancies. However, the remarkably low incidence of early pregnancy loss (3%) and the unexpected high rate of loss of clinical pregnancies (29%) in the low MEHP exposure group suggests that the positive association between MEHP exposure and early pregnancy loss and the inverse association of MEHP with loss of clinical pregnancies may also, at least partly, reflect random error because of the limited sample size.

Rodent studies support adverse reproductive effects of MEHP. Lamb et al. (1987) tested the effects of DEP, DBP, dihexyl phthalate, and DEHP on the number of litters and proportion of liveborn offspring in mice. They found that DEHP (which is rapidly metabolized to MEHP and secondary metabolites in mammals) reduced the number of litters and proportion of liveborn offspring when mixed as 0.1% or 0.3% of the diet, whereas DBP had effects at 1% of the diet and DEP had no apparent effects up to 2.5% of the diet (Lamb et al. 1987). DEHP has also been shown to induce postimplantation loss after exposure of male rats at 750 mg/kg/day (Jarfelt et al. 2005). Similarly, DBP (metabolized to MBP) has been found to act as a reproductive toxicant in female rats, where fetuses were spontaneously aborted in mid-pregnancy and hormone levels were altered in female rats exposed to 500 μg/kg/day DBP or higher (Gray et al. 2006). However, the effects in these animal studies were observed after exposures higher than the estimated daily intake for human populations, which typically is in the low micrograms per kilogram range (Frederiksen et al. 2011).

Based on animal studies, a mechanism of action of phthalates on female reproductive function can be suggested. In rats, DEHP exposure decreased the levels of estrogen and progesterone (Svechnikova et al. 2007) in addition to prolonging menstrual cycles and anovulation. These disturbances, based on *in vitro* experiments, can be explained by a DEHP-induced decrease in aromatase mRNA and protein levels, causing decreased conversion of testosterone to estradiol (Lovekamp-Swan and Davis 2003). Because low estradiol and progesterone levels have been associated with human fetal loss (Schindler 2004), the association between MEHP and pregnancy loss in our study population might reflect a phthalate-induced decrease in the secretion of these hormones.

In the conception cycle, nonsignificantly higher ORs for pregnancy loss were found at the third tertile in the conception sample for all of the measured phthalate metabolites. A tendency toward similar associations with MEHP and MEHP metabolites may be explained by a high correlation between MEHP and secondary metabolites (*r* > 0.8), but the other measured phthalate metabolites were only weakly correlated with MEHP (*r* < 0.2). Potential effects of these compounds are therefore unlikely to be explained by correlation with MEHP.

In the present study, the correlation of different phthalate metabolites between preconception sample and conception samples was between 0.08 and 0.40, indicating that individual phthalate exposure is highly variable over time (Anderson et al. 2001). Therefore, it is not surprising that we found associations between phthalate exposure and pregnancy loss only when we included the sample taken a few days before conception, because we did not expect delayed effects on implantation and embryonic development. Even when measured close to the event of interest, there could have been misclassification of the exposure due to the interday variation in the urinary concentration of these compounds (Fromme et al. 2007) and imprecision of the analysis (Hoppin et al. 2006). In our study, the coefficient of variation of concentrations obtained in quality control samples varied between 7% and 34% [see Supplemental Material, Table 1 (http://dx.doi.org/10.1289/ehp.1103552)]. Also, some variation in the ovulation day in the menstrual cycle is likely; therefore, some measurements will be closer to ovulation than others, inducing additional risk of misclassification of etiologically relevant exposure in some women. However, we do not expect this misclassification to be related to the outcome, because all samples were taken before the pregnancies occurred and analyzed without knowledge of the outcome. Therefore, this possible misclassification would most likely bias the results toward the null effect.

It can be questioned whether phthalate metabolites can be measured accurately in samples stored for several years. A recent study (Baird et al. 2010) indicated that phthalate metabolites can indeed be measured accurately on samples stored for approximately 22–24 years at –20°C. The authors reported that compared with recent samples, the stored samples contained higher concentrations, indicating no or only limited degradation, although concentrations might have been higher in the past. The correlations between metabolites and reproducibility between stored samples collected 2–4 weeks apart were similar to those of recent studies. Concentrations for a representative sample of Danish women are not available, but compared with representative samples from 60 American and 53 German women, with a concentration of MEHP of 8.3 and 10.3 ng/mL, respectively (Baird et al. 2010; Koch et al. 2003), we measured higher concentrations of MEHP and higher or similar concentrations of other phthalate metabolites. We also found a very high correlation between MEHP secondary metabolites (5-oxo-MEHP and 5-hydroxo-MEHP: *r* > 0.94) and similar correlations as in previous studies between samples collected from the same individuals at different times (Baird et al. 2010; Koch et al. 2003), indicating that our samples were unlikely to have been affected by major degradation during storage.

We included a number of potential confounders in our analysis (female age, BMI, smoking, alcohol consumption, and caffeine consumption). The self-reported lifestyle factors may have been recorded with some bias. Male exposure data were not available, which is a limitation if male exposures are also associated with spontaneous abortion and with female exposure. Sperm quality was not included as a potential confounder in the model, because it is unlikely that male semen quality is related to female phthalate exposure. Furthermore, we cannot exclude residual confounding from unmeasured potential confounders.

Our study takes advantage of the possibility of measuring exposure during a specific time window suspected to be of importance for pregnancy loss in a prospective study design. However, our study population was limited in size, and effect estimates for early pregnancy losses were imprecise. Therefore, the markedly elevated OR in this subanalysis should be interpreted with caution. The findings need to be repeated in larger cohorts before final conclusions on the adverse effects of MEHP on pregnancy loss can be made.

## Conclusions

We found associations between exposure to the phthalate metabolite MEHP in the period around conception and pregnancy loss; no associations were observed for exposures measured in the previous menstrual cycle. None of the other measured phthalate metabolites were significantly associated with pregnancy loss when measured close to conception or one cycle earlier. To our knowledge, this is the first study to show an association between phthalate exposure and pregnancy loss, and additional studies are needed to confirm these findings.

## Supplemental Material

(74 KB) PDF
