# Supplementary material for: Association between Pregnancy Loss and Urinary Phthalate Levels around the Time of Conception
Source: Environ Health Perspect. 2011 Nov 23;120(3):458–63. doi: 10.1289/ehp.1103552 (PMC3295336; doi:10.1289/ehp.1103552)
Supplement: (74 KB) PDF [file ehp.1103552.s001.pdf]

Supplementary material

Association between Pregnancy Loss and Urinary Phthalate Levels around the Time of Conception

Gunnar Toft, Bo A.G. Jönsson, Christian H. Lindh, Tina Kold Jensen, Niels H. Hjollund, Anne Vested, and Jens Peter Bonde.

Contents:

Supplementary Material, Table 1 2

Supplementary Material, Table 2 3

Supplementary Material, Table 1 : Mass spectrometric parameters, precision and detection limits for analyses of phthalate metabolites.

Compound	Mass fragment	Declustering potential (V)	Colission energy (V)	Precision[a] (%)	Detection[b] limit (ng/ml)
Monoethyl phthalate	193.1/77.2	-26	-28	11	1
Monobutyl phthalate	221.1/71.1	-26	-22	11	5
Monobenzyl phthalate	255.1/104.8	-26	-22	9	1
Monoethylhexyl phthalate (MEHP)	277.3/134.3	-26	-24	7	2
5-hydroxy-MEHP	293.3/121.0	-16	-24	10	0.2
5-oxo-MEHP	291.3/77.0	-11	-40	34	0.6
$^{2}H_{4}$-Monoethyl phthalate	197.1/125.0	-26	-17	-	-
$^{2}H_{4}$-Monobutyl phthalate	225.3/70.8	-26	-22	-	-
$^{13}C_{4}$-Monobenzyl phthalate	259.0/107.0	-26	-18	-	-
$^{2}H_{4}$-Monoethylhexyl phthalate (MEHP)	281.3/136.8	-26	-22	-	-
$^{13}C_{4}$-5-hydroxy-MEHP	297.3/123.8	-30	-25	-	-
$^{13}C_{4}$-5-oxo-MEHP	295.3/124.0	-35	-26	-	-

[a] Coefficient of variation of concentrations obtained in quality control samples analyzed in all analysis series.

[b] Concentration corresponding to three times the standard deviation of the chemical blanks

Supplementary Material, Table 2

Distribution of potential confounders according to exposure to MEHP in the cycle where women achieved a pregnancy (conception cycle). The group limits are based on tertiles of MEHP exposure in samples from the conception cycle.

Potential confounders	1st group<br>n=42<br><LOD-9.9 ng/ml	2nd group<br>n=43<br>9.9 – 22.0 ng/ml	3rd group<br>n=43<br>22.0 -64.0 ng/ml	p-value [a]
Age at enrollment in the study. Mean (min; max)	26.0 (21; 35)	25.1 (19; 32)	25.1 (20; 31)	0.44
Alcohol (drinks/week). Mean (min; max)	4.8 (0; 39)	4.6 (0; 35)	4.9 (0; 15)	0.93
Smoking (cigarettes/day) Mean (min; max)	1.4 (0; 20)	4.4 (0; 20)	1.5 (0; 17)	0.001
Caffeine intake (mg caffeine/day). Mean (min; max)	273 (25; 973)	354 (0; 1017)	289 (6; 906)	0.21
% with BMI <20.0	21.4	30.2	20.9	0.93
% with BMI 20.0-25.0	61.9	55.8	62.8	
% with BMI >25.0	16.7	14.0	16.3	

[a] ANOVA for continuous data and chi square test for categorical data.
